# Optimizing Building Rehabilitation through Nondestructive Evaluation of Fire-Damaged Steel-Fiber-Reinforced Concrete

**DOI:** 10.3390/s24175668

**Published:** 2024-08-31

**Authors:** Anastasios C. Mpalaskas, Violetta K. Kytinou, Adamantis G. Zapris, Theodore E. Matikas

**Affiliations:** 1 Laboratory of Reinforced Concrete and Seismic Design of Structures, Department of Civil Engineering, School of Engineering, Democritus University of Thrace (DUTH), 67100 Xanthi, Greece; abalaska@civil.duth.gr (A.C.M.); vkytinou@civil.duth.gr (V.K.K.); 2 Department of Materials Science and Engineering, University of Ioannina, 45110 Ioannina, Greece; matikas@uoi.gr

**Keywords:** acoustic emission, bending, compression, energy, ultrasound, steel-fiber-reinforced concrete, fire damage, building rehabilitation

## Abstract

Fire incidents pose significant threats to the structural integrity of reinforced concrete buildings, often necessitating comprehensive rehabilitation to restore safety and functionality. Effective rehabilitation of fire-damaged structures relies heavily on accurate damage assessment, which can be challenging with traditional invasive methods. This paper explores the impact of severe damage due to fire exposure on the mechanical behavior of steel-fiber-reinforced concrete (SFRC) using nondestructive evaluation (NDE) techniques. After being exposed to direct fire, the SFRC specimens are subjected to fracture testing to assess their mechanical properties. NDE techniques, specifically acoustic emission (AE) and ultrasonic pulse velocity (UPV), are employed to assess fire-induced damage. The primary aim of this study is to reveal that AE parameters—such as amplitude, cumulative hits, and energy—are strongly correlated with mechanical properties and damage of SFRC due to fire. Additionally, AE monitoring is employed to assess structural integrity throughout the loading application. The distribution of AE hits and the changes in specific AE parameters throughout the loading can serve as valuable indicators for differentiating between healthy and thermally damaged concrete. Compared to the well-established relationship between UPV and strength in bending and compression, the sensitivity of AE to fracture events shows its potential for in situ application, providing new characterization capabilities for evaluating the post-fire mechanical performance of SFRC. The test results of this study reveal the ability of the examined NDE methods to establish the optimum rehabilitation procedure to restore the capacity of the fire-damaged SFRC structural members.

## 1. Introduction

Buildings face various risks during their lifespan, with fire being one of the most destructive forces. Reinforced concrete structures are typically exposed to temperatures below 100 °C during their lifespan [1]. However, in the event of a fire, the material can reach much higher temperatures, often several hundred degrees Celsius, which can lead to severe structural damage [2,3]. The structure may remain standing even after the fire has been extinguished, presenting an opportunity to repair it rather than demolish and rebuild it, provided that it is a financially viable option [4,5,6]. In order to make that decision, it is essential to understand the mechanical behavior of concrete following exposure to these high temperatures, which enables an evaluation of the remaining strength and overall performance capabilities [7,8].

Concrete is typically resistant to thermal exposure as a result of its relatively low thermal conductivity, helping protect the reinforcing steel [9]. Nevertheless, exposure to very high temperatures for long periods, in the range of several hundred degrees Celsius or even above 1000 °C, can significantly compromise the concrete’s mechanical performance. This is caused by a combination of factors, including chemical breakdown of the structural components and thermal micro-cracking resulting from the differing expansion rates of the concrete’s constituents. Additionally, concrete may experience spalling, where the hot outer layers separate from the cooler inner layers due to increased pore pressure and temperature gradients [10]. This causes the underlying concrete to be exposed to higher temperatures, which speeds up its deterioration. When reinforcing bars or other mass reinforcement, as steel fibers become exposed to fire, after losing their protective concrete layer, they transmit heat and exacerbate the adverse consequences of high temperatures [11,12,13]. Hence, it is imperative to precisely assess the mechanical properties of the concrete following exposure to elevated temperatures in order to ascertain the feasibility of repairing the building and determine the most appropriate course of action [4,14].

Given the increasing use of mass reinforcement such as steel fibers, understanding the behavior of steel-fiber-reinforced concrete (SFRC) under fire conditions has become crucial to establish the optimal post-fire rehabilitation procedure [15]. SFRC is widely utilized in various high-performance applications, including industrial flooring, bridge decks, tunnel linings, and precast elements, due to its enhanced tensile strength, ductility, and impact resistance [16,17,18]. These applications often involve critical infrastructure where maintaining structural integrity after a fire event is essential [19]. Assessing the post-fire mechanical properties of SFRC is vital for determining whether these structures can be safely rehabilitated. Evaluating the residual strength and integrity of SFRC after fire exposure is key to developing effective rehabilitation strategies, ensuring that these structures continue to perform safely and effectively without the need for complete replacement [20]. The nondestructive evaluation techniques used in this study are not only effective for SFRC but may also be applicable to a wide range of advanced concrete materials, including emerging types of fiber-reinforced concretes, thereby broadening their potential use in structural health monitoring and rehabilitation efforts [21].

Effective rehabilitation of fire-damaged structures is highly dependent on accurate damage assessment, which can be challenging with traditional invasive methods. Common invasive techniques, such as core sampling, drilling, and destructive testing, have been widely used to evaluate the internal condition and residual strength of concrete structures. While these methods can provide detailed information on material properties, they are limited by their invasive nature, which can lead to additional damage to the already degraded structural elements. This damage not only compromises the integrity of the structure further but also limits the ability to perform continuous or repeated assessments [22,23]. In contrast, nondestructive evaluation methods, such as acoustic emission and ultrasonic pulse velocity, provide a promising alternative for evaluating the mechanical properties of fire-damaged concrete without causing additional harm [24]. Other methods, such as optical monitoring, have also been developed and explored as alternative non-contact approaches for similar purposes [25]. These nondestructive evaluation techniques have been established to be effective in the assessment of the structural state of concrete materials and structures [26]. Ultrasonic pulse velocity measurements provide insights into concrete’s elastic properties and internal conditions, allowing correlations to be drawn with its strength and overall integrity [27,28]. Similarly, acoustic emission monitoring employs surface-mounted piezoelectric sensors to detect the elastic waves generated by internal cracking events, enabling a reliable assessment of the material’s fracture behavior [26]. The analysis of acoustic emission indices can yield valuable information about the damage content and strength characteristics, as well as help identify the dominant modes of fracture occurring within the concrete [29,30,31]. While time-domain parameters, such as the rise-time-to-amplitude ratio (RA), average frequency (AF), and amplitude, have proven effective in evaluating damage and fracture processes in concrete, it is important to note that frequency-domain parameters, such as peak frequency and centroid frequency, also play a crucial role in damage analysis. Several studies [32,33] have demonstrated that frequency-domain features can provide valuable information regarding the nature and progression of damage within materials as these parameters are highly indicative of specific damage modes, such as crack initiation, stable crack growth, and rapid crack propagation. Additionally, measuring dissipation energy through techniques such as vibrodine analysis has been proposed as a method for detecting cracks within concrete structures [34].

In the field of thermally damaged concrete, researchers have observed that the relationship between the maximum temperature and residual strength is not straightforward. However, several studies have reported that the concrete’s remaining strength can drop by 50% to 80% when exposed to temperatures of 800 °C or higher [35,36,37,38]. Similarly, the strength of the concrete–steel bond was found to reduce significantly at temperatures of around 450 °C [39]. Additionally, the elastic modulus of the thermally damaged concrete decreases in a comparable manner, while its toughness or capacity to absorb energy during fracture is also reduced by a factor of two or more [14,40]. Interestingly, despite the reduction in strength, the thermally damaged concrete often exhibits higher ductility [11,14,41], a characteristic that could be valuable in certain applications.

Ultrasonic pulse velocity is a valuable technique for assessing the condition of concrete that has been exposed to high temperatures during a fire [42]. This method can help determine the depth of the damaged layer within the concrete and provide an estimate of the remaining structural strength [43]. The technique is sensitive to changes in the material’s stiffness caused by the exposure to elevated temperatures [9,12,28,44]. Studies have shown that wave attenuation, measured in the low-megahertz frequency range, increases significantly as a function of the temperature that the concrete has experienced [45,46,47]. Furthermore, the dispersion curves at these frequencies exhibit a substantial shift towards lower velocity levels for concrete exposed to temperatures below or slightly above 500 °C [46,47]. Researchers have also utilized nonlinear wave techniques to monitor the progressive damage in thermally affected concrete [46,48].

The use of acoustic emission monitoring has a more limited applicability when evaluating the properties of thermally damaged concrete [49]. AE techniques have been employed to track the cracking behavior of various materials exposed to extreme heat, such as in open flame conditions [50]. Specifically for concrete, studies have shown that the thermal micro-cracking required to generate detectable AE signals does not typically occur until the material reaches temperatures above 180 °C [11]. However, a significant increase in the number of recorded AE events has been observed as the temperature rises from 500 °C to 750 °C [50]. This increase in AE activity has enabled researchers to develop predictive models of the spalling behavior of concrete exposed to severe fire conditions [51,52].

Furthermore, when thermally damaged concrete specimens are mechanically tested, the release of AE energy is found to occur at a slower rate compared to undamaged concrete [11]. This slower AE response aligns with the more ductile nature of the fire-affected material, which can accommodate greater maximum strains prior to failure. Moreover, the width of the fracture process zone, as measured by AE source location techniques, has been observed to increase in thermally damaged concrete [41]. Finally, AE monitoring of concrete specimens containing embedded metal reinforcement, subjected to increasing temperatures up to 600 °C, has demonstrated the technique’s sensitivity in detecting the deterioration of the concrete–steel bond caused by fire exposure [4].

In this study, the response of steel-fiber-reinforced concrete (SFRC) specimens to direct fire exposure, rather than the more common method of elevated temperatures in an oven, was investigated. Conducting this type of experiment poses significant challenges due to the specialized equipment, test chambers, and safety precautions required. Nevertheless, the facilities of a brick manufacturing industry were utilized to carry out the investigation. After exposing the concrete samples, containing 2% steel fibers per volume, to temperatures of 850 °C, the specimens were evaluated using ultrasonic interrogation and mechanical testing in compression and bending, while simultaneously applying acoustic emission monitoring. The findings provide valuable insights into the mechanical response of SFRC after fire exposure. Notably, a discrete difference was observed in the acoustic emission characteristics between sound and fire-damaged specimens, an observation that has not been reported previously. This difference results in interesting correlations with the ultimate mechanical properties of the SFRC affected by fire. The integration of data obtained from acoustic emission and ultrasonic pulse velocity techniques can provide a more accurate assessment of the residual strength of SFRC that has been exposed to significant thermal damage.

## 2. Experimental Procedure 

### 2.1. Materials and Experimental Setup

The experimental procedure builds upon a preceding research project [53], mirroring the testing setup previously executed by the same research team. This continuation entails adjustments tailored to the current investigation’s objectives and parameters.

For the SFRC, a mix was formulated, comprising fifteen samples: nine allocated for bending tests and six for compression, respectively. The specimens included prismatic beams measuring 100 × 100 × 400 mm, used for four-point bending tests, while the other types were cubes sized 150 × 150 × 150 mm, designated for compression testing. The aggregate composition consisted of 56% crushed sand, 13.87% fine gravel, and 30.13% coarse gravel, with a maximum aggregate size of 31.5 mm. The water-to-cement ratio was maintained at 0.70 by mass. The characteristics of water absorption and density of the constituents were as follows: crushed sand exhibited a density of 2601 kg/m^3^ with a water absorption rate of 0.98%, fine gravel displayed a density of 2621 kg/m^3^ with a water absorption rate of 0.75%, and coarse gravel demonstrated a density of 2681 kg/m^3^ with a water absorption rate of 0.61%. The cement utilized adhered to the type CEMII/A-M(P-LL) specifications. The specific mixture details are described in Table 1.

The concrete exhibited a bulk density of 2359 kg/m^3^, with the ambient temperature maintained at 25 °C. Workability, as determined by the slump test, yielded a result of 11 cm. Specimens were subjected to curing in water infused with calcium hydroxide at a controlled temperature of 23 ± 2 °C. The mean compressive strength recorded at three (3) days was *f_c_*_(3)_ = 18.0 MPa; at seven (7) days was *f_c_*_(7)_ = 24.7 MPa; and at twenty-eight (28) days was *f_c_*_(28)_ = 33.8 MPa, accompanied by a standard deviation of 3.0 MPa.

**Figure 1:**
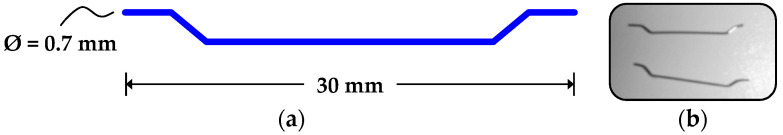
Hooked-end fibers H2_30 2% per volume: (**a**) schematic representation of the steel fiber; (**b**) photograph of the actual steel fibers as used.

Meanwhile, steel fibers designated as H2_30 were utilized for reinforcement. They are characterized by hooked-shaped edges, as can be seen in Figure 1. They have a diameter of 0.7 mm, a length of 30 mm, and are added at 2% per volume in the mixture.

**Figure 2:**
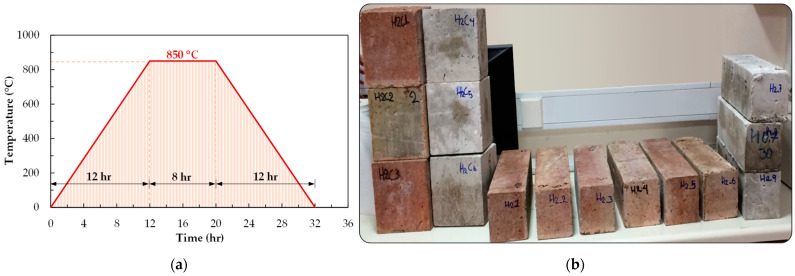
(**a**) Fire exposure sequence; (**b**) healthy and fire-damaged bending and compression specimens.

Subsequently, a total of nine (9) specimens were exposed to fire, six (6) for bending and three (3) for compressive testing. The fire-induced deterioration occurred within the same furnace for compression and bending specimens. The total number of specimens tested for the mechanical properties is presented in Figure 2.

Thermal degradation necessitates specialized apparatus and is not invariably a straightforward undertaking to execute. To conduct this study, the fire exposure was carried out in a specialized facility. More specifically, the thermal degradation process took place inside a firing chamber at a brick production site. The specimens were exposed to direct heat on all sides apart from the side on which they were resting. Compression and bending specimens were subjected to fire with a peak temperature of 850 °C. The thermal exposure progressed incrementally to the peak temperature over a duration of 12 h, following which the temperature was maintained constant for a further 8 h (Figure 2a). Although utilizing standardized fire curves in experimental studies that simulate fire scenarios is typical, the furnace used in this experimental setup could not maintain the stringent conditions that a standard fire curve would typically demand. The primary objective of this study was to describe and explain the differences between healthy and fire-damaged concrete specimens while ensuring a consistent fire-loading pattern across all tested specimens. This procedure has been successfully accomplished, providing valuable insights into the behavior of SFRC under the specific fire conditions employed.

#### 2.1.1. Bending Test Setup

**Figure 3:**
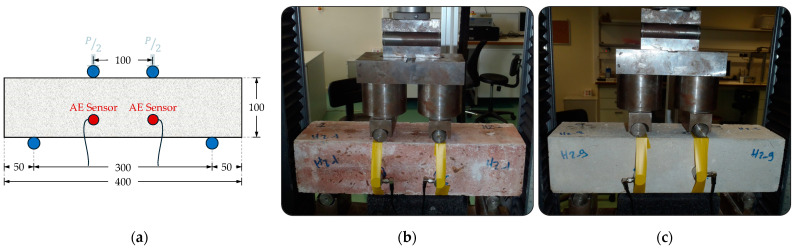
Bending test setup accompanied by simultaneous AE monitoring: (**a**) schematic representation of the bending test setup; (**b**) fire-damaged SFRC specimen and (**c**) healthy SFRC specimen.

Six of the prismatic beam specimens, having been exposed to a temperature of 850 °C, underwent four-point bending tests, as can be seen in Figure 3a, employing a span length of 300 mm. The testing procedure utilized a servohydraulic Instron Model 5967 (30 kN capacity) (INSTRON^®^, Norwood, MA, USA). The loading and support apparatus adhered to the specifications outlined in BS EN 12390-5:2000 [54]. Loading on the specimen was applied without eccentricity or torque. A 0.12 mm/min displacement rate was maintained throughout the testing process. Furthermore, three intact healthy prismatic bending specimens, as shown in Figure 3b, were tested with the same experimental setup to assess the flexural behavior of the SFRC specimens, with concurring AE monitoring.

#### 2.1.2. Compression Test Setup

**Figure 4:**
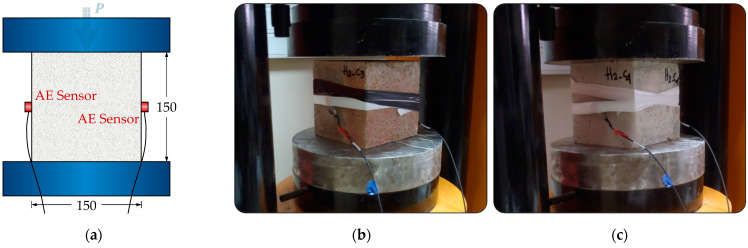
Compression test setup accompanied by simultaneous AE monitoring: (**a**) schematic representation of the compression test setup; (**b**) fire-damaged SFRC specimen and (**c**) healthy SFRC specimen.

A total of six samples were subjected to compression testing. This included three specimens that had been exposed to a temperature of 850 °C, as illustrated in Figure 4a, and three undamaged specimens, as depicted in Figure 4b. The samples were loaded at a constant rate of 166.7 Newtons per second (Nt/s) until failure. The test was automatically concluded once a decrease in the applied load was detected.

### 2.2. Monitoring Methods and Procedure 

#### 2.2.1. AE

**Figure 5:**
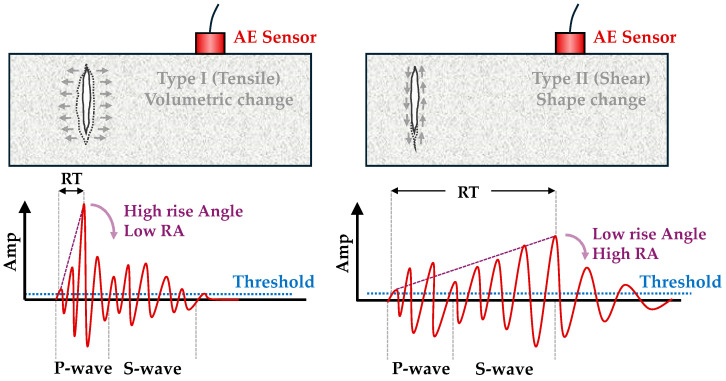
Typical AE waveform.

Two piezoelectric sensors with resonance frequencies of 150 kHz, specifically the R15 type from Physical Acoustics Corp. (PAC), were utilized for acoustic emission (AE) measurements. The signals were converted into digital form at a sampling rate of 3 MHz using a PCI-8 card from PAC, following pre-amplification by 40 dB. To enhance acoustic coupling, a layer of grease was applied between the sensors and the surface of the sample, and the sensors were affixed in place using tape throughout the experiment, as depicted in Figure 3 and Figure 4. Only signals exceeding an amplitude of 40 dB were recorded, while signals lacking sufficient energy were disregarded. Figure 5 illustrates a typical acoustic emission waveform alongside a description of its key parameters.

The primary characteristics determined from the received waveform include amplitude (A), which represents the magnitude of the cracking occurrence, and energy (ENER), which corresponds to the area under the rectified signal envelope and the energy released by the crack [29]. Furthermore, it has been established that the RA value and the frequency content of the waveform (represented as threshold crossing over duration) offer insights into the fracture mode. This frequency content is quantified as the average frequency (AF), calculated as the ratio of the total number of threshold crossings (or counts) to the duration of the acoustic emission signal. The AF provides insights into the fracture processes within the material, where higher AF values are typically associated with rapid energy release events like crack propagation or brittle fracture, and lower AF values often indicate slower processes such as plastic deformation or micro-crack growth. These metrics exhibit significant correlations with mechanical properties and are explored alongside the accumulated hit activity in subsequent sections [53]. Particularly in this study, alterations in the primary waveform parameter values were delineated throughout mechanical loading.

#### 2.2.2. Ultrasonic Inspection

**Figure 6:**
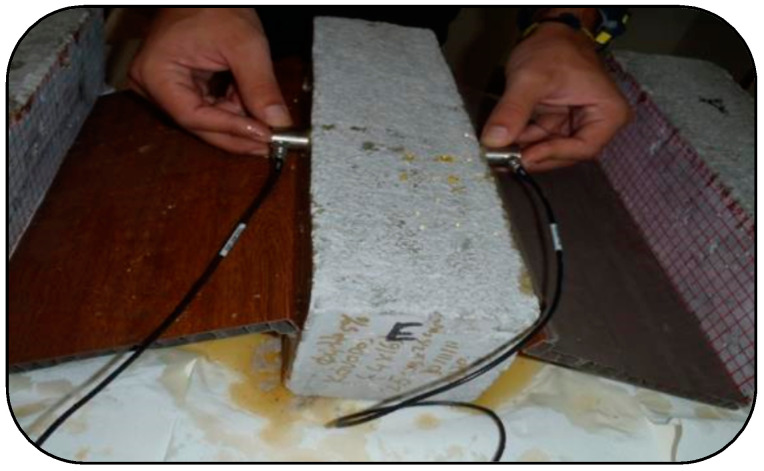
UPV test on prismatic bending steel-fiber-reinforced concrete specimen.

Before conducting the fracture testing, all specimens underwent ultrasonic interrogation along their longitudinal axis, as illustrated in Figure 6. The distance between the pulser and the receiver was set at 100 mm for the bending specimens and 150 mm for the compression specimens, respectively. Three measurements were obtained along different vertical wave pathways for bending specimens, whereas two measurements were recorded for compression specimens. For each specimen, the average of these measurements’ values was calculated. The resonance frequency of the transducers was set at 150 kHz (R15, Mistras Group, Athens, Greece). A Tektronix AFG3102 (Testwall Ltd, Dublin, Ireland) waveform generator was employed in conjunction with a two-channel PCI-2 Mistras board data acquisition system. The generator provided excitation with one cycle of 150 kHz to the pulser, synchronized with the acquisition board to serve as a reference. The ultrasonic pulse velocity (UPV) was determined by measuring the wave propagation time delay to the first detectable disturbance of the received waveform. The findings were averaged for each specimen [9].

## 3. Results

The fundamental properties derived from the mechanical and UPV testing are summarized in Table 2. All specimens showed a significant reduction in maximum load-bearing capacity. This reduction was accompanied by a noticeable decrease in ultrasonic pulse velocity when compared to intact specimens. Detailed results for both bending and compression tests are thoroughly discussed below. Furthermore, Table 3 outlines the basic properties of AE monitoring during mechanical testing for both fire-damaged and healthy bending specimens. Table 4 presents the mean values for each compression specimen, categorized by fire-damaged and healthy conditions.

**Figure 7:**
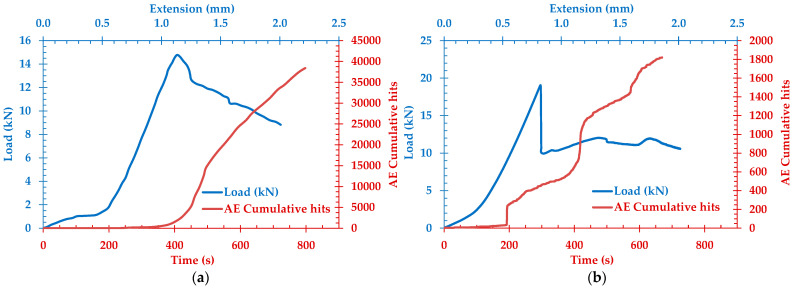
AE cumulative hits and bending load history for (**a**) fire-damaged SFRC specimen and (**b**) healthy SFRC specimen.

The load history and the cumulative AE activity curves for the bending tests are shown in Figure 7. Case (a) corresponds to fire-damaged and (b) to healthy SFRC specimens. Besides the variation in maximum load resulting from the degradation of the heated specimen, there is a noticeable difference in the AE behavior. Regarding the degraded specimen, the gradual rise in AE rate suggests the progressive development of micro-cracking throughout the loading process, as inferred from the increased AE activity commonly associated with crack formation and propagation in weakened concrete matrices [30,53]. This extensive micro-cracking is a result of the weakened matrix due to thermal damage, which causes continuous damage accumulation as the load increases. On the contrary, for the intact specimen with steel fiber reinforcement (SFR), there is a period characterized by almost a vertical increase in AE activity, corresponding to the onset of macro-cracking and significant load drop. In addition, the total cumulative emission is much higher in the fired-damaged specimens in comparison with the intact ones, and this behavior is similar to a previous study with different types of fibers [53].

More specifically, within undamaged healthy specimens, the shift between the initial phase at the early loading stage and the fracture initiating is abrupt; AE led to a sharp escalation in hits occurring at the fracture point. This observation suggests a decline in the brittleness of fire-damaged SFRC, coupled with a substantial 80% decline in strength.

In instances involving the degraded specimen, the augmentation in AE rate exhibits a progressive and uniform progression, characterized by a continuous escalation over temporal intervals. In contrast, for the intact SFRC specimen, a discernible phase of abrupt AE rate escalation emerges, synchronizing with the onset of the initial load decrement. This temporal occurrence aligns with the manifestation of macro-cracks within the specimen.

**Figure 8:**
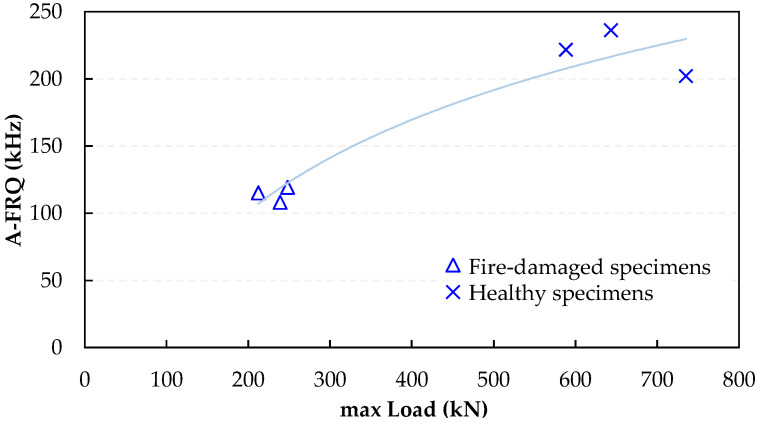
Analysis of AF versus maximum load in compressive tests of fire-damaged and healthy specimens.

**Figure 9:**
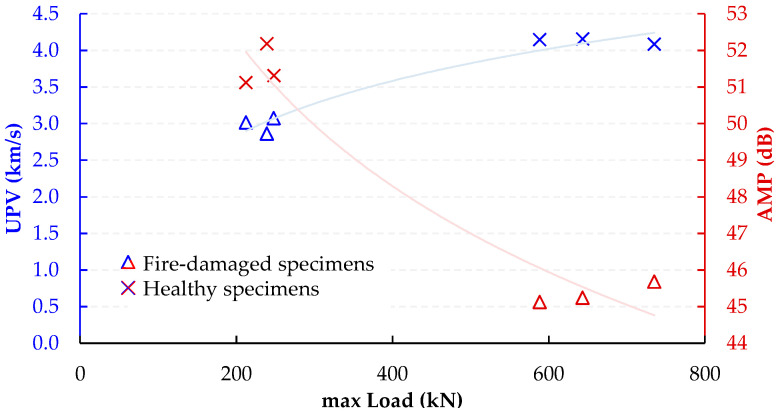
Correlation of mean AE amplitude, UPV, and compressive strength for fire-damaged and healthy SFRC specimens.

**Figure 10:**
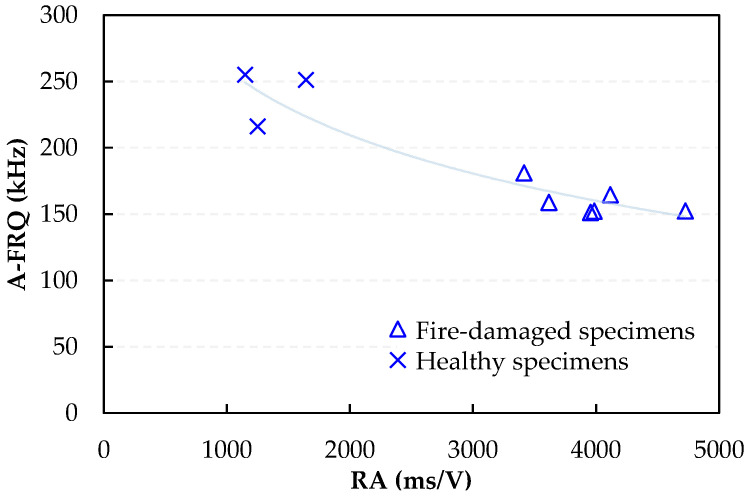
Correlation between amplitude frequency (AF) and rise-time amplitude (RA) for bending tests on fire-damaged and healthy SFRC specimens.

In addition to the overall emission count, waveform metrics such as amplitude (AMP), average frequency (AF), and rise time to amplitude (RA) offer vital information on the structural condition and fracture process. Comparative analysis was performed on these key parameters for both healthy and fire-damaged SFRC, with average values calculated throughout the testing period. In Figure 8, Figure 9 and Figure 10, logarithmic fitting curves are shown, which were applied to establish the trend between the healthy and fire-damaged specimens. This approach was primarily utilized for illustrative purposes, with the main objective to highlight the uptrend or downtrend in AE parameters between healthy and damaged specimens under identical loading conditions. The logarithmic fit provides a clear visual representation of the differences in AE behavior due to fire damage, aiding in the comparative analysis of the specimen groups. Figure 8 shows the correlation between the AF and maximum load for specimens under compression, including both fire-damaged and healthy samples.

There is a clear and noticeable distinction between the two groups. Healthy compression specimens exhibit higher strength and higher AF values. On the other hand, the specimens that were damaged by fire display a decrease in compressive strength and significantly lower AF values from all emitted signals. This clear segregation between the clusters indicates significant disparities in primary AE parameters between healthy and fire-damaged specimens. Figure 9 presents a detailed comparison of the mechanical and elastic characteristics of compressive specimens that have been damaged by fire and those that are in a healthy condition. The healthy compression specimens exhibit higher strength and higher AF values. In contrast, the fire-damaged specimens show a marked reduction in compressive strength and significantly lower AF values from all emitted signals. This clear segregation between the clusters highlights significant disparities in primary AE parameters between healthy and fire-damaged specimens. The correlation between AE mean amplitude, UPV, and compressive strength for these specimens further illustrates these differences.

Figure 10 extends this analysis to bending specimens, revealing a strong correlation between AF and RA values. The decrease in AF values with increasing RA in bending specimens further supports the findings of the compression tests, indicating consistent patterns of damage and fracture behavior under different loading conditions.

**Figure 11:**
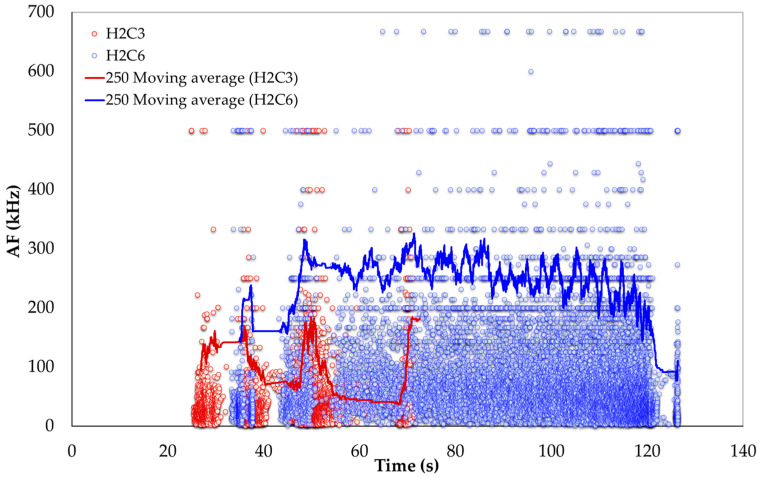
AF versus time for fire-damaged (H2C3) and healthy (H2C6) SFRC specimens under compression, with 250-value moving averages.

**Figure 12:**
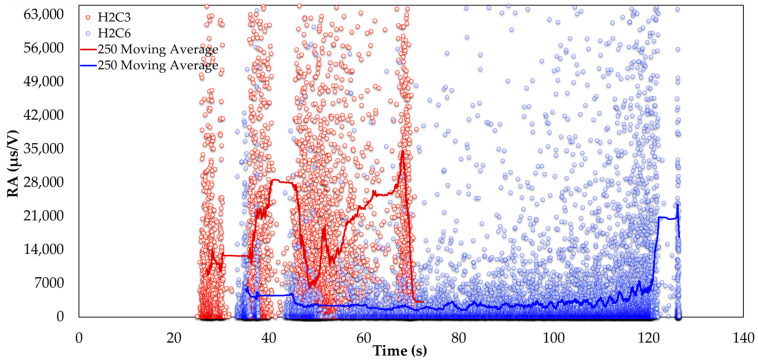
RA versus time for fire-damaged (H2C3) and healthy (H2C6) SFRC specimens under compression, with 250-value moving averages.

**Figure 13:**
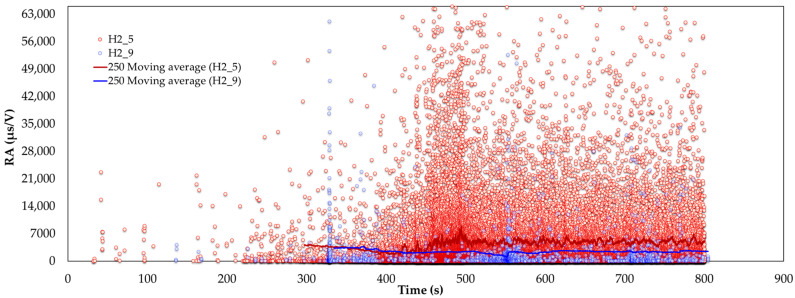
RA versus time for fire-damaged (H2_5) and healthy (H2_9) SFRC bending specimens, with 250-value moving averages.

The well-documented relationship between compressive strength and ultrasonic pulse velocity in the existing literature [20,26,53] is further validated by this experimental study. In addition, a strong correlation between compressive strength and the average amplitude of AE during testing was observed. These findings are crucial for assessing fire-damaged structures, as AE measurements can be integrated into minimally invasive procedures, such as drilling or capo-tests [55,56], to provide valuable insights into the extent of fire-induced damage. Previous studies [57] have utilized AF and RA to characterize the progression of damage in cementitious materials. Figure 11 and Figure 12 present two-dimensional plots of AF and RA versus time for the compressive tests on SFRC. Furthermore, Figure 13 presents the AF versus time for the bending specimens, offering additional insights. These plots highlight the study’s primary innovation, which involves monitoring the AE response during both compression and bending tests on fire-damaged SFRC specimens. This approach allows for the characterization of damage degradation and the monitoring of structural integrity under different loading conditions.

Figure 11 illustrates the acoustic frequency (AF) versus time for both fire-damaged (H2C3) and healthy (H2C6) SFRC specimens, with trends shown using 250-value moving averages. The data indicate that the fire-damaged specimen (H2C3) consistently shows lower AF values compared to the healthy specimen (H2C6). This reduction suggests a diminished capacity of the fire-damaged material to sustain high-frequency AE waveforms, likely due to microstructural degradation caused by fire. Furthermore, the variability in AF values for the fire-damaged specimen reflects ongoing internal damage processes, contrasting with the relatively stable AF values seen in the healthy specimen.

Figure 12 and Figure 13 present the rise time to amplitude (RA) values over time for fire-damaged and healthy SFRC specimens, each with 250-value moving averages. In the results, fire-damaged specimens (H2C3 in Figure 12 and H2_5 in Figure 13) exhibit higher and more variable RA values compared to healthy specimens (H2C6 in Figure 12 and H2_9 in Figure 13). Elevated RA values indicate the occurrence of damage events, such as cracking.

The substantial fluctuations in RA values for the fire-damaged specimens highlight the increased severity and frequency of internal damage caused by fire exposure. In contrast, the healthy specimens maintain consistent and lower RA values, indicating their structural integrity is largely intact. 

In general, analyzing the AF and RA values in Figure 11, Figure 12 and Figure 13 reveals a coherent pattern of degradation in fire-damaged specimens. The fire-damaged specimens exhibited lower AF and higher RA values compared to the healthy specimens, indicating a shift in the fracture behavior due to the structural degradation caused by high temperatures. High temperatures cause thermal decomposition of the cementitious matrix, leading to micro-crack formation. These micro-cracks alter the fracture mechanics by reducing the material’s stiffness and bond strength. As a result, crack propagation in fire-damaged specimens becomes more gradual with less energy released, leading to lower AF values. The increased rise time of AE signals due to the extensive network of crack paths contributes to higher RA values. This change in failure mode is caused by thermal degradation, reduction in stiffness, increased ductility, and the formation of voids, all of which are consequences of fire exposure. These AE parameters are effective in detecting and quantifying the extent of damage, offering a nondestructive means of evaluating the structural health of SFRC.

## 4. Conclusions

This study investigated the impact of severe thermal damage on the mechanical behavior of SFRC specimens using nondestructive evaluation techniques, specifically acoustic emission and ultrasonic pulse velocity. The study was aimed at enhancing our understanding of fire-induced damage and its implications for the rehabilitation and maintenance of reinforced concrete structures.

Through experimental testing, SFRC specimens containing 2% per volume steel fibers were exposed to direct fire at a peak temperature of 850 °C. The specimens were then subjected to bending and compression tests, with concurrent AE monitoring. The primary objective was to demonstrate that AE parameters, such as amplitude, cumulative hits, and energy, exhibit strong correlations with mechanical parameters and damage caused by fire. In addition, this study aimed to characterize the structural integrity of the specimens during loading by analyzing the distribution of AE hits and changes in AE parameters. The following conclusions are drawn:

- This study found a significant reduction in maximum load-bearing capacity and UPV values for all fire-damaged specimens compared to intact specimens. This indicates substantial structural degradation due to exposure to fire.
- AE monitoring revealed distinct differences between healthy and fire-damaged specimens. Fire-damaged specimens exhibited higher RA and lower AF values, indicating an increased severity and frequency of internal damage. The variability in AE parameters over time highlighted the progressive nature of the damage.
- A strong correlation between compressive strength and the average amplitude of AE signals was observed, confirming the effectiveness of AE as a diagnostic tool. Similarly, the well-documented relationship between compressive strength and UPV was validated.
- The strong correlations between AE parameters and mechanical properties, as well as between UPV and strength, highlight the effectiveness of these nondestructive evaluation techniques in assessing the structural integrity of fire-damaged SFRC.
- The inferred extensive micro-cracking in fire-damaged specimens, suggested by the gradual rise in AE activity, emphasizes the need for thorough evaluation of internal damage. This interpretation is consistent with established studies using AE data to monitor micro-crack development in fire-affected concrete [49,51,53]. This micro-cracking, which accumulates progressively, contrasts with the abrupt damage observed in healthy specimens and highlights the complexity of assessing fire-damaged SFRC.

The results of this study highlight the potential of integrating AE and UPV techniques into the assessment and rehabilitation of fire-damaged SFRC structures. The distinct differentiation in AE parameters between healthy and damaged specimens underscores the value of AE monitoring as a minimally invasive diagnostic tool that can provide valuable information on the extent of fire-induced damage without further compromising the structure. By combining AE and UPV techniques, it is possible to achieve a more precise characterization of the remaining strength in SFRC subjected to severe thermal damage, thereby facilitating more effective rehabilitation and maintenance strategies. Moreover, the capability of the examined NDE methods to identify the extent of damage and guide optimal rehabilitation procedures underscores their critical role in restoring the structural capacity of fire-damaged SFRC members.

## 5. Recommendations for Future Studies

The current study has demonstrated the effectiveness of nondestructive evaluation (NDE) techniques, such as acoustic emission (AE) and ultrasonic pulse velocity (UPV), in assessing the mechanical behavior of fire-damaged steel-fiber-reinforced concrete (SFRC). Building on these findings, several avenues of future research can be pursued to deepen our understanding and broaden the applicability of these techniques. Some potential key areas of research could include the following:

- Combining time-domain and frequency-domain parameters could lead to a more comprehensive assessment of structural integrity across various concrete structures, enhancing the accuracy and reliability of damage evaluations.
- Utilizing advanced techniques like scanning electron microscopy (SEM) to investigate microstructural changes due to fire exposure could provide deeper insights into the mechanisms underlying AE and UPV behaviors, applicable to a wide range of concrete types.
- Future studies should develop predictive models that integrate AE and UPV data with temperature exposure history to predict the remaining service life of fire-damaged structures, aiding in rehabilitation decisions.
- Research on different types of fiber reinforcements, such as glass or synthetic fibers, could identify the most effective materials for fire-resistant applications and provide insights into how various reinforcements influence residual strength and damage mechanisms.
- Future research should explore the integration of traditional invasive assessment methods with NDE techniques like AE and UPV. Comparative analyses between these approaches could also quantify their respective benefits and limitations, potentially leading to more effective and less damaging assessment strategies for concrete structures.

## Figures and Tables

**Table 1:** The exact steel-fiber-reinforced concrete mix proportions.

Cement (Type II 42.5 N)	Water	Crushed Sand	Fine Gravel	Retarder–Plasticizer (CHEM I)	Retarder–Plasticizer (CHEM II)
(kg/m^3^)	(kg/m^3^)	(kg/m^3^)	(kg/m^3^)	(kg/m^3^)	(kg/m^3^)
350	245	1161	404	1.54	1.96

**Table 2:** Mean values of strength and UPV per different batch per different mechanical test.

	Average Max Load (kN)	UPV (m/s)
Fire-Damaged Bending Specimens	15.45	3233.29
Healthy Bending Specimens	24.70	4274.10
Fire-Damaged Compression Specimens	233.20	2982.07
Healthy Compression Specimens	655.72	4127.07

**Table 3:** Mean values of the AE parameters per bending specimen.

*Specimen Name*	*Hits*	*Sum ENER* (10^−14^ V^2^s)	*ENER* (10^−14^ V^2^s)	*DURATION* (μs)	*AMP* (dB)	*A-FRQ* (kHz)	*RA* (μs/V)
*Fired-Damaged Bending Specimens*							
*H2_1*	38,339.00	802,196.00	20.92	884.35	47.51	152.20	4726.96
*H2_2*	28,362.00	708,930.00	24.99	865.51	47.79	164.46	4117.19
*H2_3*	28,110.00	460,369.00	16.38	706.18	47.75	158.75	3618.20
*H2_4*	43,890.00	920,812.00	20.98	796.78	48.08	152.13	3985.14
*H2_5*	23,365.00	371,693.00	15.91	698.94	47.31	180.90	3414.55
*H2_6*	47,296.00	821,251.00	17.36	718.71	47.72	151.15	3955.37
*Average*	34,893.67	680,875.17	19.42	778.41	47.69	159.93	3969.57
*Healthy Bending Specimens*							
*H2_7*	272.00	3393.00	12.43	342.71	46.41	216.01	1248.72
*H2_8*	3463.00	30,357.00	8.76	313.54	46.02	255.16	1146.57
*H2_9*	7852.00	43,661.00	5.56	281.97	45.78	251.10	1640.35
*Average*	3862.33	25,803.67	8.92	312.74	46.07	240.76	1345.21

**Table 4:** Mean values of AE parameters per compression specimen.

*Specimen Name*	*Hits*	*Sum ENER* (10^−14^ V^2^s)	*ENER* (10^−14^ V^2^s)	*DURATION* (μs)	*AMP* (dB)	*A-FRQ* (kHz)	*RA* (μs/V)
*Fired-Damaged C* *ompression* *Specimens*							
*H2_c1*	3641.00	2,384,061.00	654.60	17,210.64	51.31	119.26	14,388.27
*H2_c2*	4305.00	2,000,598.00	464.61	12,167.51	51.12	115.06	12,171.61
*H2_c3*	5934.00	1,501,131.00	252.93	9513.33	52.18	107.97	14,883.35
*Average*	4626.67	1,961,930.00	457.38	12,963.83	51.54	114.09	13,814.41
*Healthy C* *ompression* *Specimens*							
*H2_c4*	21,845.00	421,591.00	19.30	970.37	45.68	202.09	5878.04
*H2_c5*	13,137.00	372,592.00	28.36	1481.19	45.12	221.61	7293.30
*H2_c6*	13,777.00	355,977.00	25.84	1010.75	45.24	236.18	3686.38
*Average*	16,253.00	383,386.67	24.50	1154.10	45.35	219.96	5619.24

## Data Availability

The data supporting the findings of this study are available from the corresponding author upon reasonable request.
